# Music as a collaborating actor: new insights into the nature and role of music in psychedelic-assisted psychotherapy

**DOI:** 10.3389/fpsyt.2025.1541528

**Published:** 2025-04-30

**Authors:** Justin Dwyer, Robert B. Johnston, Clare O’Callaghan, Voula Kallianis, Margaret L. Ross

**Affiliations:** ^1^ Department of Psychosocial Cancer Care, St Vincent’s Hospital (Melbourne), Melbourne, VIC, Australia; ^2^ School of Business, University College Dublin, Dublin, Ireland; ^3^ Department of Medicine, The University of Melbourne, Parkville, VIC, Australia; ^4^ Department of Social Work, Caritas Christi, St Vincent’s Hospital, Melbourne, VIC, Australia

**Keywords:** music therapy, psychedelic assisted psychotherapy, end-of-life, palliative care, psilocybin, psychotherapy

## Abstract

**Background:**

Music has been identified as a central feature of psychedelic-assisted psychotherapy (PAP) and has hitherto been understood to amplify the psychedelic experience in a predictable way that has been codified into music recommendations and playlists.

**Purpose of study:**

To re-evaluate the nature and role of music within the participant’s world during psychedelic-assisted psychotherapy.

**Method:**

Phenomenological analysis of participants’ descriptions of music during a randomised control trial of PAP at end of life involving two doses and a semi-structured interview following each dose.

**Findings:**

Music undergoes a profound change during PAP that radically transforms it from everyday recorded music into a series of internally generated multisensory and deeply personal experiences that arrive fully formed and are instantly known by the participant. Some of these are constituted into actors that collaborate with the participant and the psychotherapist in their ongoing psychotherapy endeavours. This stands in stark contrast with the everyday properties of music described by those in the placebo group.

**Conclusions:**

An alternate understanding of music in PAP is suggested that radically departs from the view that music is “administered” as part of PAP. There are profound implications for the practise of PAP and further research.

**Clinical trial registration:**

Australian New Zealand Clinician Trials Registry identifier, ACTRN12619001225101.

## 1 Introduction

Psychedelic-assisted psychotherapy (PAP) involves the judicious use of a psychedelic compound nested within a structured psychotherapy process and is an active research arena across multiple conditions including mood and anxiety disorders, substance abuse, chronic pain, and distress associated with end of life (1). The psychedelic compound is typically ingested in a curated environment that supports introspection in the presence of the treating psychotherapists (2), and music has a central role in these treatment sessions, where it acts as a catalyst of emotion, connection, and moments of transcendence (3, 4). Music is now embedded in treatment and research PAP protocols (5, 6) and is at the intersection of a lively dialogue between psychotherapists, neuroscientists, musicologists, and anthropologists (7, 8).

The first systematic evaluation of the role of music in PAP examined the differential impact of five different music “treatment conditions” after Lysergic acid diethylamide (LSD) ingestion, comparing no music, miscellaneous music, familiar music with and without headphones, and unfamiliar music (9). The authors concluded that music was an “important and influential part of the treatment milieu” and affirmed the importance of personalised music selection. Bonny and Pahnke (10) built upon this in their influential paper that described the phases of the psychedelic experience from the pre-onset of the drug effect to the peak psychedelic experience and then resolution and the corresponding music that best supported this process (10). Detailed and evocative descriptions of the music are provided, such as “very powerful, strongly structured music with insistent rhythms and a wide frequency range” (10), to help guide music selection, and these have been formulated with later contributions into recommendations for playlists and criteria for the selection of music (11, 12).

This categorisation of music in PAP according to properties such as melody and tempo that can be reliably sequenced to maximise therapeutic effects (11) is mainly supported by small cross-sectional surveys of participant experience and the views of experts in the field (3). Opinions vary on the importance of standardised or personal music in PAP (10, 13) and the role of other individual factors such as trauma and the effects of colonisation (14).

Thus, work so far has viewed music as an important component of the larger *treatment environment* that is provided to the participant and, in many respects, is characterised by researchers as either a non-specific amplifier of the psychedelic experience (13) or as a substantial entity with controllable properties administered by the practitioner along with psychedelic medication (11). Against this background, our research asks, “How might music be more productively characterised in the light of how it is actually experienced within the participant’s lifeworld^1^ during PAP?”

We found that participants report that music undergoes a radical change during PAP, and this finding necessitates a total re-evaluation of the nature and role of music in PAP with immediate implications for psychotherapists working in PAP.

## 2 Method

Addressing the question of how music is experienced within the participant’s lifeworld requires an approach that prioritises the participant’s perspectival descriptions and, therefore, demands a phenomenological approach to ensure that the complexity and nuance of individual experience are adequately captured. Thus, we adopted an interpretive phenomenological approach aimed at “attaining an understanding and proper description of the experiential structure” (16, p. 9) that proceeds entirely from the lived experience of the participants. Our research stance was informed by our work as clinicians and psychotherapists in palliative care services in keeping with other phenomenological research contexts that focus on in-depth lived experiences (17).

We drew upon a large, longitudinal qualitative dataset to ensure a breadth of responses, which allowed us to contrast the music experience of those with a placebo group and differential music experiences across two doses. A reflexive process of interviewing and iterative refinement through a grounded theory framework informed our thematic analysis (18). There was no *a priori* hypothesis about the experience of the participants, and the aim was not merely to elicit how music was experienced but, rather, how it became part of the participant’s lifeworld during and even after the PAP.

### 2.1 Setting

This report is part of a larger, recently completed randomised control trial with an open-label extension of PAP using psilocybin for anxiety and depression at the end of life. The open-label extension was given 6 weeks after the placebo-controlled dose to ensure that every participant was offered the active drug. The study setting was a major general hospital in a large Australian city, and participants were recruited via health professional referral and self-referral. Enrolment commenced in January 2020 and ended in January 2023 after several pauses due to local COVID-19-related regulations.

### 2.2 Study design

Inclusion criteria were 18–85 years old, living with life-limiting illness and experiencing psychological distress associated with their illness, Australia-modified Karnofsky Performance Status (AKPS) 50+ score (19), able to tolerate oral psilocybin, and approved by the treating specialist. Exclusion criteria were known central nervous system involvement; current or historical diagnosis meeting DSM-5 (20) criteria for Schizophrenia, Psychotic Disorder (except substance or medically induced) or Bipolar I or II disorder, or who have a first-degree relative with these diagnoses; current or 5-year history of DSM-5 criteria for alcohol or drug dependence (except caffeine and nicotine); meets DSM-5 Criteria for Dissociative Disorder, Anorexia Nervosa, Bulimia Nervosa; medical and psychological factors considered incompatible for establishing rapport or safe exposure to psilocybin ingestion; and unable to give informed consent.

The intervention consisted of a manualised program of outpatient psychotherapy provided by a psychotherapy dyad. Each psychotherapy session was 120 min in duration and was scheduled on three successive days. There were three psychotherapy sessions before the randomised control trial (RCT) dose that focused on the following:

- Understanding the experience of the terminal illness and its broader impact on everyday life.
- Crafting a formulation of the participants’ experience and hopes for participating in the trial that drew upon formative developmental, relationship, spiritual, and medical themes.
- Development of therapeutic rapport.
- Educational component focused on common effects of psilocybin, navigating challenging experiences, and managing boundaries.
- An experiential component that provided an orientation to the psychedelic dosing space and music.
- Engagement with a support person.

On the double-blinded RCT psychedelic dose day, participants ingested either niacin 100 mg or psilocybin 25 mg early in the morning and then lay down and donned an eye mask and noise-cancelling earphones in keeping with previous studies (21, 22). They listened to their chosen music selection as part of their experience and remained in the dose space with the therapists until judged medically and psychologically appropriate to leave with their support person at the end of the day.

Following the RCT dose, there were three sessions of psychotherapy. The first of these sessions took place the day immediately after the psychedelic dose day and then 3 and 6 weeks later. Each of these sessions was 120 min in duration and was aimed at understanding and consolidating any insights or changes that emerged (23), and the open-label dose was prepared 6 weeks later after which another three sessions of psychotherapy followed.

### 2.3 Design of music component

A music history was obtained of preferences and dislikes across the lifespan as part of a psychotherapy assessment and formulation, and then participants were offered an experience of three playlists they could choose from as part of the orientation to the psychedelic dosing space. The playlists were the Johns Hopkins Psychedelic Research Unit (duration 6 hr 5 min), Mendel Kaelen Full Playlist 1.3 (duration 5 hr 35 min), and a playlist of our own design (duration 7 hr 51 min) devised by four of the psychotherapists (a music therapist, a psychiatrist and a psychologist both with backgrounds as musicians, and another psychiatrist) under the guidance given by Bonny and Pahnke (10), Kaelen (13), and Barrett et al. (5).

On the psychedelic dose day, the therapists were able to modify the playlist in response to the wide variation in the onset of the drug effect. The physiological consequences of serious medical illness resulted in some individuals entering peak experience soon after ingestion, whilst others were delayed by up to 3 hr. The therapists could also reflexively adapt the music in response to the unfolding therapeutic process in keeping with the advice of Bonny and Pahnke (10) and Garcia-Romeu (21). The therapists were all experienced palliative care clinicians with their own extensive psychotherapy training and specific training for this trial.

### 2.4 Data collection

There were three qualitative interviews conducted by a trained qualitative researcher by phone or face-to-face and then transcribed verbatim. These interviews were conducted the day before the RCT psychedelic dose day, 3 weeks after the RCT dose day, and 3 weeks after the open-label dose day. Music-related questions were informed by previous research on music experience in psychedelic therapy (13) and began with “How do you think music affected your experience, if at all?” Additional questions were “How did the different styles of music affect your experience?”, “Please describe the music you preferred and why you think that is so?”, and “Please describe the music you did not prefer and why you think that is so?” followed by probes to expand and qualify responses. This report was drawn from 57 interviews comprising 41 post-psilocybin ingestion and 16 post-niacin ingestion sessions. The mean PD1 interview (n = 30) length was 24.8 min. The mean PD2 interview (n = 27) length was 35.2 min.

### 2.5 Data analysis

Demographic data are presented using percentages and absolute numbers in **Table 1**. All interview data were coded (text segments were given researcher-created descriptive labels). Coded music-related data was then identified for further analysis. A total of 388 codes relating to participants’ experience with music were separately examined, and their content was summarised into researcher-created categories and then researcher-created themes. A qualitative inter-rater reliability procedure was followed (24), and all analysts reached a consensus with the findings. Overall, data were analysed for patterns related to how participants experienced music related to the PAP trial and described how music affected their trial experiences and the mechanisms through which these effects were perceived to occur (25). The ATLAS/ti 9 (26) qualitative data management software was used. Study reporting was guided by the Consolidated Criteria for Reporting Qualitative Research (COREQ) (27). Data interpretations were not checked with participants to mitigate the burden, as many died after the study follow-up period, and there is no evidence that participant feedback on research findings improves their quality (28, 29).

**Table 1:** Participant characteristics.

Demographics	Placebo n = 17	Active n = 18
Age years (SD)	51 years (SD 14.5)	61 years (SD 19.4)
Gender female %	55%	60%
Current partner	56%	76%
Prior psychotherapy	72%	88%
Prior psychedelic exposure	27%	29%

Primary medical illness		
Malignant/non-malignant	13/4	16/2

Primary psychiatric diagnosis by absolute number		
Major depressive disorder	8	7
Adjustment disorder with mixed depressed mood and anxiety	3	5
Adjustment disorder with depressed mood	3	2
Adjustment disorder with anxiety	1	1
Generalised anxiety disorder	2	0
Persistent depressive disorder	1	0
Panic disorder with agoraphobia	0	2

### 2.6 Ethical considerations

This study was approved by the St Vincent’s Hospital Human Research and Ethics Committee and was registered with the Australian New Zealand Clinician Trials Registry (ACTRN12619001225101). All participants provided informed consent prior to enrolment.

### 2.7 Participant enrolment

One hundred six potential participants were screened, and 71 were excluded. Fifty-one were excluded at phone intake, and a further 20 were excluded after signing informed consent on account of disease progression (n = 13), abnormal serology (n = 4), deceased during the screening process (n = 1), psychiatric exclusion (n = 1), and inability to travel to the trial site (n = 1). Thirty-five participants were randomised. Twenty-eight participants completed the two doses, with seven participants withdrawn from the psilocybin group across both doses on account of disease progression (n = 2), resuming antidepressant treatment (n = 1), death due to underlying disease (n = 1), anxiety about a subsequent dose (n = 1), family commitments (n = 1), and COVID-19 travel restrictions (n = 1). All placebo group participants completed two doses.

## 3 Findings

### 3.1 Theme 1: Metamorphosis

Music undergoes a change so astonishing that participants may not recall having heard any music at all. Complex multi-instrumental arrangements are broken down into singular forms, such as an isolated drum or solitary tone, that surge towards the participant. In this elemental form, music fuses with other experiences, such as an embodied sensation and a key memory, and these metamorphic complexes emerge fully formed from within the participant’s lifeworld.

#### 3.1.1 Music undergoes a metamorphic shift

There is a fundamental shift in the way music is perceived and an uncanny quality that is completely at odds with the everyday experience of listening to music. For some, the magnitude of this shift can be so great that it loses all resemblance to music, and even the same playlist from an earlier dose session becomes unfamiliar.

it’s probably worth noting that I didn’t have any perception of hearing music while I was under the effects of the drug… (P34).

I suppose the unexpected was, the music didn’t seem the same as it was. I was hearing, probably hearing different things or different tones. I don’t know what it was, but I can’t remember hearing some of the music that I heard the second time. I couldn’t remember hearing it the first time… (P10).

#### 3.1.2 Music is experienced in elemental form

Music is experienced as a vein of musical elements crystallised out from the full recorded work. These disembodied rhythms and instruments ebb and flow within the participant’s lifeworld, and this constant flux of elemental music can have an unsettling effect.

… the strumming repetition, I suppose that that was disturbing about it and, and it didn’t feel melodic in any way. It just felt disturbing … it reinforced the sense of of fragmentation and disconnection, because each each piece was quite solitary… (P5).

#### 3.1.3 Symphonic moments of experience

Elemental music is recalled in symphonic experiences involving the totality of the participant’s lifeworld that go far beyond the simple sensory crossover of synaesthesia. These moments bring embodied experience, emotion, and key moments in the personal narrative into vivid relationships that are instantly recognisable, and with the following properties.

*Symmetry of experience*—moments involving elemental music cascade across all senses in a symmetrical and coherent manner.

And the music was in sync with the imagery that my mind was creating, and I was in sync with the music to the point that my body was, in sync and vibrating to the sound and the visual. So much so that I, I was overcome with just this intense emotion of, wht I was looking at (P12).

*Simultaneity*—elemental music comes into the moment that the participant is experiencing a sense of being instantaneously and fully involved. There is no direction of cause and effect, nor any sense of the passage of time.

then the flowers would open as a stringed instrument would play and they would play at the same you know all these flowers were opening at the same time as bits of layers of the music so then the music was completely inside the experience as well like everything would shift and flow and change as the music changed (P22).

#### 3.1.4 Elemental music emerges from within the participant’s lifeworld

The moments involving elemental music are not heard; rather, they emerge fully formed from within the participant’s lifeworld. This sense of internal generation is evident in a sense of having created the music, loss of awareness of music as something separate from self, and in moments of immersion in music that had never been experienced before.

I remember at one point thinking, have I stopped listening to the music that is on the headphones, because I like I was controlling the music and I was like. I was like, Well, I can’t be listening to the track because the music is moving with me. And I’m like, in-time with me and um. So I thought maybe, maybe I had like subconsciously stopped listening to, you know, the actual soundtrack and I was making up the music in my head. Like. I was like one with the system so to speak (P6).

#### 3.1.5 Comparison with placebo experience

These changes contrast with accounts of the everyday properties of music, such as volume and contextual memories given by those who had received a placebo.

There was one particular piece stood out to me, not because I found it to be relaxing or, you know, particularly entrancing. It was just simply because it sounded like a song that I heard in Bali once when I, well when I traveled to Bali before… (P8).

There was one exception within the placebo group of a participant who described vivid experiences during the placebo dose. Of note, this participant had rich spiritual views about life and very strong expectations related to receiving the active drug. Following the open-label dose 6 weeks later, this participant had no recollection of hearing any music at all.

… although like the music was like obviously like prevalent, I was so deep in my trip that, you know,.that’s why I can’t remember any music (P33).

### 3.2 Theme 2: Quickening

The participant’s lifeworld becomes animate and brims with encounters that carry the full impact of a live interaction. Alloyed into these moments of contact, elemental music gives voice to loved ones, ancestral communities, and others. Amidst the jostle, elemental music is encountered as a *sentient presence* with its own intentions for the participant.

#### 3.2.1 Elemental music gives voice to encounters with loved ones

Loved ones are given form and made immediately recognisable in the presence of elemental music. There were opportunities to reconnect, to separate oneself out, and to repair damaged relationships with early life figures who were brought into the present moment within the elemental music experience.

some of the music I remember evoked the not memory, but just the the notion of letting go of my children and not seeing them again….Other music was just like, at one point I saw my husband, who passed away six years ago. And so that sort of moments of just sheer joy (P29).

#### 3.2.2 Ancestral communities and other figures emerge from elemental music

Ancestral communities appeared with elemental music and were galvanised into action that involved the participant. Elemental music became a shared language that joined the participants in these communities. Disturbing figures with darker motives emerged for some in concert with elemental music.

that was fantastic that was just like when there was just an entire all of the cultures and the people in their regalia we come together and we were celebrating and we were in this big jungle kind of um wanting to dance (P22).

think that was the part that I was really scared, frighten and that music really bothering me. It was also the voice …. Even with the music you can direct and do anything you can get and from the other hand the voice, another voice was telling me that you can ask what you want…. But and the other voice was saying that see even if you want, they won’t listen to you (P26).

#### 3.2.3 Elemental music as a “sentient” actor

Elemental music itself was encountered as a sentient actor that had authority within the participant’s lifeworld. This could be experienced in a benevolent way through elemental music sharing knowledge with the participants about themselves or through more tyrannical experiences of a destructive nature.

The music was its own entity, and it was trying to kill me (P27).

I felt very connected to the music and the music felt alive and like it had a life of its own and I was part of it (P3).

### 3.3 Theme 3: Apprenticeship

Elemental music is woven into the surging metamorphic experiences and striking encounters that flood the participant’s lifeworld. This brings the participants into direct contact with aspects of their personal story, and the therapists, in an emotionally charged atmosphere. Elemental music is intimately involved in both the creation of these moments and the ever-evolving array of psychological tools needed to deal with them.

#### 3.3.1 Learning to work inside elemental music

Elemental music is integral to the ebb and flow of teeming metamorphic complexes within the participant’s lifeworld. Harrowing experiences of elemental music can emerge that test the limits of endurance and push the participant to find creative solutions. Elemental music can also confer a reassuring sense of safety that gives the participants the freedom to explore and learn about themselves.

Each song felt like a different sort of lesson or a different journey. Like I was very aware that I’d sort of go in and I’d be getting all these insights and then the song would sort of come to an end, and then it’d be like, oh that’s done. And there’d be a little gap, and then the next one would start and I’d dive into the next lesson (P25).

I wish this music with drums would stop. You know, like, I felt that that was kind of really mixing up in my mind and it was all going fast to and unpleasant….then I’m like you know, that I thought no. Let’s get through this (P24).

#### 3.3.2 Elemental music as an emerging psychotherapist

Elemental music focused the participant on important personal stories spanning early life adversity, unhealthy relationship patterns, and other sources of psychological distress. In concert with elemental music, these deeply meaningful moments were able to be experienced differently, and some reframed these in a way that revealed previously unseen strengths and resources.

… that the sense of dislocation, dissociation, fragmentation have always been the great fears that I’ve carried in my life. So in a sense, all it was, was bringing out some fairly unpleasant experiences (P5).

… one that was very much more like a drum beat, felt very strong, and I had sort of a experience where I was sort of like a warrior for my family, but it was definitely very tied to the strong beat and drumming. And in the, the music sort of strained up a little bit more and felt a little bit stronger in my chest. That definitely felt like it was super closely related to what I was learning and a lot of them, I don’t really remember the actual music, I just remember the scene or the experience itself (P25).

#### 3.3.3 Learning about the therapists through elemental music

For some participants, elemental music became a channel of direct communication by which the therapists were brought into the participant’s lifeworld. Participants felt that events unfolding within their experience were known by the therapists when elemental music seemingly changed in direct response to the dominant emotional undercurrent. This back and forth between participant and therapist maintained the constant sense of therapist engagement.

[the therapists] were able to control some of my feelings through the music, because, I mean, I assume they were able to change music when they thought I needed it, it needed to be changed or something like that (P14).

then at one time, I thought I felt like they are putting this music on just to leave me just to take me in I felt as like a big brother controlling thing (P24).

#### 3.3.4 Elemental music as an arena to develop mastery

For those participants who received two doses of psilocybin, elemental music became the proving ground in which once-troubling experiences could be revisited and dealt with in an entirely new way. A sense of accomplishment was immediately recognised, as the same moments were dealt with differently from dose to dose.

the first session I had I was fighting the music because I didn’t like it. So I was trying to pull it back to what I did like … Whereas the second one. The second study, the music. I let it go and just followed the music (P14).

this time I felt like I had a mastery of the music (P24).

### 3.4 Theme 4: Collaboration

Elemental music takes its place within the participant’s lifeworld as a collaborating actor in shared psychotherapeutic endeavours. The focus of this collaboration includes everyday emotional management and deeper insight-oriented work surrounding the events that transpired during the psychedelic dose and in early life.

#### 3.4.1 Elemental music is established within the participant’s lifeworld

Elemental music takes root in the participant’s lifeworld and now suffuses everyday experiences of music with the same symphonic qualities as it had on the psychedelic dose day. The participant is primed for elemental music experiences and can bring these to the fore through the intentional use of recorded music.

…music. I just love music. My music is on 24/7. And I just float with music and like, I can put music on now and close my eyes and just float…. Yeah. I can just lose myself in music (P10).

….use the music that I’ve been given to make my own self guided meditation about the experience so that I can keep it, keep it going and yeh revisit it (P24).

#### 3.4.2 Collaborating with elemental music in everyday self-care and connection

The participants work with elemental music to deepen their sense of connection to others and to moments in which their own emotional needs are acknowledged.

…it’s after the house is quiet and that’s when I can put that music on I’ll put on the most beautiful candles I’ve got that gorgeous scent on because they sent me home with some of the scented oils so that would be a kind of time when I would listen to it, I’ve listened to it once or twice just lying down but that that’s not as easy to find the time to do, my little boy spends the night, a couple of nights a week at his dad’s so they’re often times when I sort of engage in a bit of ritual (P22).

#### 3.4.3 Collaborating with elemental music to explore the dose day experience

Participants could work with the elemental music in a joint effort to make sense of partially understood moments from their dose day that felt unresolved.

…when I listen to the music now, it brings back memories of what happened in the sessions … I feel as if I’m making more sense of it now. Especially after the second [dose] (P10).

[I’m] spending up to 2 hours a day doing meditation with the soundtrack. Trying to recreate the state. So even still being aware that there is a darkness or a blackness or a void or emptiness somewhere that I’ve still to resolve (P17).

#### 3.4.4 Collaborating with elemental music to gain insights into foundational experiences

Elemental music collaborated in the shared exploration of important early life experiences, connecting the past and the present in a way that generated fresh insights. The complex, symphonic nature of elemental music made sense of early life experiences that had been embedded on an emotional rather than a verbal level, and the therapists could join this process alongside elemental music.

It certainly has triggered some recollections of past events. I find the music has mostly been very peaceful and relaxing, although a couple of times bits of the music, I found you know elevated heart rate and, you know, a bit of stress coming out (P34).

As I’ve explained to [therapists] … some of the music was extraordinarily disturbing and recurring, so I can still hear that some of that in my head. And that’s not very pleasant … that’s Okay, because I think in the discussion afterwards with [therapist] the reason that that had such difficulty for me is that the sense of dislocation, dissociation, fragmentation have always been the great fears that I’ve carried in my life (P5).

## 4 Discussion

Against the dominant view of music as an externally administered substance (5, 13), in this project, we sought to uncover how music presented in the participant’s actual experience during PAP and, consequently, how the nature and role of music in PAP can be more productively understood.

We found that music underwent a profound and irreversible alteration within the participant’s lifeworld during PAP. The transition was from recorded music originating from an external source to elemental music that was generated *within* the participant as a symphonic complex with other aspects of the immediate moment being experienced and in a manner that was instantaneously understood. In this form, music could be encountered as a collaborating actor and then consolidated into an enduring ally for ongoing psychotherapeutic work. This contrasts sharply with the perspectives of the placebo group who described the very properties identified as important by earlier authors, such as music ordering, volume, and style (10, 11). This shift was dramatically illustrated by P33, where high expectations of a powerful psychedelic trip contributed to a vivid experience with music in the placebo arm, whilst during the open-label dose 6 weeks later, music was completely transformed and unrecognisable in comparison. Together, these findings indicate that it is inaccurate to assume that music is encountered during PAP as it is generally understood outside of these sessions and risks the therapists being misled when working with music-related therapeutic material that has emerged during PAP.

On a conceptual level, the music is no longer part of the therapeutic environment in the same vein as low lighting and aesthetic décor (30). It is known by the participant as intimately as their own breath. This calls for an entirely different orientation by the therapists from simply providing music to seeing themselves working alongside music. This way of working with music finds support in the guided imagery and music field, where therapists set out to intentionally forge a relationship with music that is therapeutic and enduring (31, 32). This changed relationship to music needs to acknowledge the life-long place of music for the participant as a continuous thread that links formative memories with ongoing therapeutic work with music that will continue long past the end of therapist involvement.

The presence of music within the participant’s lifeworld is so different from that of the same music as experienced by an external observer that our findings do not support the assertions made by some authors about the use of operationalised criteria for the curation of playlists (11, 12). Rather, we conclude that therapists need to remain actively engaged with the presence of music through the PAP process, being aware of dominant instruments and rhythms and transitions in the music, all the while remaining attuned to the participant’s emotional state and holding the person’s whole story in mind to prompt later exploration of the experience.

All of this requires the therapists to develop their own relationship with the music *with each participant* in the same open and curious way as they consider each participant’s story.

Many of the participant experiences included in reports by other authors resonate with this alternate understanding of music. Participants in Gaston’s seminal work described the music as seeming to “control or lead my emotions” and that “it helped to get a deeper feeling of what’s going on in my mind” (9, p. 17), and Bonny and Pahnke provided many evocative quotes such as “….part of the notes in a particular melody assumed human stature, which I recognized as that of my parents, although they actually never appeared as people …” (10, p. 3). Our approach to the role of music during PAP was inspired by the work of these authors and others (13, 33). However, their findings are underpinned by an implicit view of music as a controllable “substance” *provided by* the therapy team to the participant, and this is reflected in their recommendations regarding music programming and presentation. Beginning with a phenomenological approach to describing participant experience, our findings completely upend this perspective. We have found that the nature and role of music within the participant’s lifeworld during PAP depart radically from how the same music is experienced and provided by the treating therapists. Furthermore, this finding has immediate implications for practice.

### 4.1 Implications for clinical practice

From a clinical perspective, there are multiple opportunities for therapists to deepen the impact of the therapy by working with the music in the way that is experienced by the participants throughout the entire PAP process, as follows.

#### 4.1.1 Assessment

Seeking out formative experiences of music and sound can begin to focus the attention of the participant and therapists on what may emerge. Memories of parents singing from childhood, or the deafening silence of unspoken family conflict, are examples of important moments that may resonate within experiences of elemental music. For the therapists, cultivating a curiosity about these sound memories is essential (34) and may involve obtaining pieces of music, listening to recordings of identified nursery rhymes, and through this beginning to locate music as a presence within the participant’s lifeworld. Understanding the participant’s musical preferences, both liked and disliked, is an important part of the assessment especially where prior trauma is linked with music (14).

#### 4.1.2 Orientation to the process

Providing an experience of music that attunes the participant to the metamorphic process and the presence of elemental music can be part of preparation. Here, music needs to be sensed in an alloy of experience, and the use of drone notes, overtones, and non-Western instruments as well as exercises such as humming with music may be helpful (35). As important is the language used to describe music—the emphasis here is not just on what the music does or helps to access but rather the importance of learning to work with elemental music—this shift in language ascribes agency to the participant *and* music and primes the participant for the emergence of this therapeutic ally.

#### 4.1.3 During the psychedelic dose session

On the psychedelic dose day, we would agree with Bonny and Pahnke (10) and others on the view that the music selected should be personalised for the participant (13), and this requires that the therapists are familiar with a repertoire of music that can be flexibly adapted. In our trial, this was mandated by the unpredictable onset of drug action in a cohort of participants with all manner of serious medical illnesses. When noting a response to music, it is important to remember that the patients are not listening to the same music as the therapist. Rather, they are experiencing something that is internally generated in elemental form only and in complex with other important things that may be fully alive in the participants’ lifeworld. The focus is on this unique opportunity to learn how to work and then collaborate. Statements such as “trust, let go and be open” (36) and “in and through” (37) may have the unintended effect of relegating the participants to a passive and receptive role that inhibits their sense of agency in the ongoing psychotherapy work.

The consolidation of elemental music as a collaborator can occur during the psychedelic dose session or in the aftermath. Here, the participants revisit important and formative stories from their lives equipped with the music. The therapists need to welcome this into their sessions and may even present music that the participants need to access certain knowledge of themselves and understandings of their psychedelic dose day. The language used around music here needs to explicitly acknowledge this different relationship to music and to speak about it with the respect that is accorded to a learned colleague.

Much of this work can take place without reference to the arbitrary delineations of preparation, dosing, and integration and, in doing so, standing back and honouring the person’s life story over all else.

The integration of music and sound in this way during PAP requires a skilled music therapist in the team who can work as a therapist with participants and provide much-needed practice wisdom and therapeutic skills to the entire team. From a practical perspective, a reliable sound system with noise-cancelling headphones and external speakers, a library of music, and access to instruments such as sound bowls for vibroacoustic experiences are recommended.

### 4.2 Implications for research

The need for therapists to be responsive to music is often reported (10, 13), and this is deserving of further study that may complement and extend our findings. Understanding how music choices are made in these moments and how therapy dyads reach an agreement and monitor the impact of changing the music during and after a participant’s psychedelic experience is a corresponding gap in our knowledge about working alongside music during PAP.

A related research question is on the development of therapeutic competency with music in PAP therapists and how this may be taught in the same way as other aspects of PAP, such as dealing with challenging experiences.

A key recommendation would be for qualitative methodologies to be embedded within all PAP studies given the potential to inform practice.

### 4.3 Ethical implications

The extraction and decontextualisation of psilocybin from Indigenous knowledge-holders is an uncomfortable truth in the development of contemporary PAP, and there are calls for engagement with Indigenous communities at all levels of the “psychedelic renaissance” (38). Discussions around the use of music in PAP are an opportunity for cultural exchange with Indigenous people who acknowledge the place of sacred music in ritual and ceremony and can contribute to the developing relationship across treatment and research settings (39). We would argue that Indigenous music should only be used in PAP settings in the context of a relationship with its traditional custodians.

### 4.4 Limitations and opportunities

Whilst small, our study generated the largest qualitative dataset in PAP to date and is strengthened by an RCT design that allows for the comparison of placebo and active experiences, and interviews at different timepoints that permit a more nuanced exploration of participant experience. The clinical cohort studied was participants at the end of life, a unique population segment with high levels of personal (40) and family psychological distress (41). Whilst many of our participants met diagnostic criteria for major depression and anxiety disorders, there may be differences in general population cohorts that add to our understanding of the role of music. Replicating this work with non-English speaking and Indigenous populations would further strengthen our findings.

## 5 Conclusion

Music has been regarded as a key part of the therapeutic milieu in PAP but previously has been characterised as an external and controllable influence with known properties that can be tweaked for effect (10, 11, 13, 30). We sought to more fully describe the experience of music within the participant’s lifeworld during PAP. Taking a phenomenological approach and using selected grounded theory techniques, we found that music is irreversibly altered within the participant’s lifeworld and is likened to a sentient actor capable of collaborating with both participant and therapists in service to the psychotherapy process. Adopting this alternate understanding of the nature and role of music in PAP will make possible radically new ways of working as a psychotherapist in PAP.

## Data Availability

The datasets presented in this article are not readily available because qualitative data set. Requests to access the datasets should be directed to drjustindwyer@gmail.com.

## Footnotes

1 Lifeworld (15, p. 132) here and later refers to the participant's phenomenal world—the world as it appears to an engaged person in a particular way of life. Note that viewing the participants' experience during PAP as a part of their lifeworld is integral to our phenomenological approach.
